# The HSP90 inhibitor KW-2478 depletes the malignancy of BCR/ABL and overcomes the imatinib-resistance caused by BCR/ABL amplification

**DOI:** 10.1186/s40164-022-00287-w

**Published:** 2022-05-27

**Authors:** Dachuan Zeng, Miao Gao, Renren Zheng, Run Qin, Wei He, Suotian Liu, Wei Wei, Zhenglan Huang

**Affiliations:** 1 Department of Clinical Hematology, Key Laboratory of Laboratory Medical Diagnostics Designated by the Ministry of Education, School of Laboratory Medicine, Chongqing Medical University, Chongqing, China; 2 Department of Laboratory Medicine, The First Affiliated Hospital of Chongqing Medical University, No. 1, Youyi Road, Yuzhong District, Chongqing, 400016 China

**Keywords:** BCR/ABL, HSP90 inhibitor, KW-2478, Malignancy, Imatinib resistance

## Abstract

**Background:**

With the widespread clinical application of tyrosine kinase inhibitors (TKIs), an increasing number of chronic myeloid leukaemia (CML) patients have developed resistance or intolerance to TKIs. BCR/ABL is the oncoprotein of CML. HSP90 is an essential chaperone of BCR/ABL and plays an important role in protein folding and the function of BCR/ABL. Therefore, inhibiting the chaperone function of HSP90 may be an effective strategy for CML treatment and to overcome TKI resistance.

**Methods:**

The effect of KW-2478 on CML cell viability, apoptosis and cell cycle progression was detected by CCK-8 assay or flow cytometry. The levels of BCR/ABL, HSP90 and other signalling proteins were detected by western blots. The mitochondrial membrane potential was detected by flow cytometry combined with JC-1 staining. The interaction between BCR/ABL and HSP90α was detected by coimmunoprecipitation. The effect of KW-2478 on BCR/ABL carcinogenesis in vivo was investigated in CML-like mouse models.

**Results:**

KW-2478 inhibited growth and induced apoptosis of CML cells. KW-2478 inhibited the chaperone function of HSP90α and then weakened the BCR/ABL and MAPK signalling pathways. This treatment also caused an increase in p27 and p21 expression and a decrease in cyclin B1 expression, which led to G2/M phase arrest. The mitochondrial pathway was primarily responsible for KW-2478-induced apoptosis. KW-2478 had a synergistic effect with imatinib in growth inhibition. Notably, KW-2478 had a stronger effect on growth inhibition, apoptosis induction and cell cycle arrest of K562/G01 cells than K562 cells. KW-2478 could effectively prolong the mouse lifespan and alleviate disease symptoms in CML-like mouse models.

**Conclusions:**

This finding demonstrated that KW-2478 had anticancer properties in imatinib-sensitive and imatinib-resistant CML cells and illustrated the possible mechanisms. This study provides an alternative choice for CML treatment, especially for TKI-resistant patients with BCR/ABL amplification and TKI-intolerant patients.

**Supplementary Information:**

The online version contains supplementary material available at 10.1186/s40164-022-00287-w.

## Background

Chronic myeloid leukaemia (CML) is a myeloproliferative tumour caused by haematopoietic stem cells. The pathogenesis of this disease involves the translocation of chromosomes 9 and 22 [1]. The BCR/ABL oncoprotein encoded by the *BCR/ABL* oncogene maintains constitutive tyrosine kinase activity, induces downstream signalling pathway activation, promotes CML cell proliferation and inhibits cell apoptosis [2]. According to statistics, the number of CML cases increased from 31.8 thousand in 1990 to 34.2 thousand in 2017 [3]. Although the disease burden of CML has declined globally from 1990 to 2017, treatment of CML remains difficult [4]. In the history of CML therapy, many therapeutic methods have been used in CML treatment, such as cytarabine, hydroxyurea, allogeneic stem cell transplantation, and IFN-α. These methods have improved the symptoms and prolonged the survival of patients to a certain extent [5]. However, it was not until the advent of tyrosine kinase inhibitors (TKIs) targeting the ATP binding site of BCR/ABL in 2001 that significant progress was achieved in CML treatment [6]. There are also some novel therapeutic strategies, such as using Ab@Tf-Cou6-PLGA NPs as intracellular transporters of antibodies to degrade BCR/ABL oncoproteins in CML cells [7]. Nonetheless, with the widespread clinical application of TKIs, an increasing number of CML patients have developed resistance or intolerance to TKIs. TKI resistance mechanisms have been identified and are divided into two categories: BCR/ABL-dependent and BCR/ABL-independent mechanisms. Mutation or amplification of BCR/ABL leads to BCR/ABL-dependent TKI resistance. ABC transporter-mediated drug efflux and a resistance-promoting microenvironment contribute to BCR/ABL-independent TKI resistance [8]. Two second-generation TKIs, dasatinib and nilotinib, were approved in the United States and Europe from 2006 to 2007 as second-line treatments for CML patients resistant to or intolerant of previous treatment (including imatinib). As a third-generation TKI, ponatinib was approved in the United States in 2012 for CML patients with the T315I mutation [9]. Studies have also shown that imatinib resistance can be modulated by long noncoding RNAs that target miRNAs, thereby changing the expression of targets associated with drug resistance [10]. Moreover, most CML patients are dependent on TKIs throughout their life and will soon relapse once the drug is withdrawn [11, 12]. Therefore, it is urgent to develop alternative treatments [13].

BCR/ABL is the key oncogenic protein for CML. Chaperone proteins are important for BCR/ABL to exert its oncogenicity, and HSP90 is one of the vital chaperone proteins for BCR/ABL [14, 15]. Although HSP90 family proteins are ubiquitously expressed in normal cells, many oncoproteins expressed in mutant forms show greater dependence on HSP90 for their stability and activity than their normal counterparts, e.g., BCR/ABL, EGFR and Raf1 [16]. The HSP90 level is low in normal cells but highly elevated in CML cells [17]. BCR/ABL is an important client protein of HSP90 that protects BCR/ABL from degradation via the ubiquitin-proteasome pathway. However, HSP90 inhibitors prevent BCR/ABL from binding to HSP90 [18]. Therefore, targeting HSP90 to inhibit its chaperone function for BCR/ABL may be an effective strategy for CML treatment. A variety of HSP90 inhibitors have been investigated for CML therapy, including geldamamycin (GA) derivatives and radicicol (RD) [18, 19]. The compound 17-allaminso-17-demethoxygoldamycin (17-AAG) is a GA derivative. It has been reported that 17-AAG can downregulate BCR/ABL expression and then induce K562 cell apoptosis [20]. RD also decreased BCR/ABL in CML cells [21]. However, due to their physicochemical properties and safety profile, the therapeutic application of these early HSP90 inhibitors is limited [22, 23]. For example, the water solubility of 17-AAG is poor [24], while RD derivatives cause severe toxicities in mice [25].

KW-2478 was shown to be a novel resorcinol-based HSP90α inhibitor with good water solubility and few side effects. Much more importantly, KW-2478 has strong antitumor effects on several malignancies, such as multiple myeloma (MM), B-cell malignancies and liver cancer, even in MM patients with tolerance to melphalan and patients with relapsed/refractory B-cell malignancies [26–29]. Given the prominent antitumor effect and good safety performance of KW-2478 and the importance of HSP90 in maintaining the structure and function of BCR/ABL, we wondered whether KW-2478 would be an ideal therapeutic method for CML patients. Moreover, amplification of BCR/ABL is one of the TKI-resistant mechanisms. Compared to its normal counterpart c-ABL, BCR/ABL is significantly more reliant on HSP90 for stability and activity. Therefore, we wondered whether KW-2478 could decrease the malignancy of BCR/ABL and overcome TKI resistance induced by BCR/ABL amplification.

In this study, we used imatinib-sensitive K562 and imatinib-resistant K562/G01 cells as the main research objects. Imatinib resistance in K562/G01 cells depends on BCR/ABL amplification rather than point mutation. It has been reported that the *BCR/ABL* fusion gene is amplified and that the BCR/ABL fusion protein shows increased kinase activity in K562/G01 cells [30]. We explored the effects of KW-2478 on CML cells in vitro and in vivo and the possible mechanisms exerted by KW-2478. Furthermore, we investigated whether KW-2478 could increase the sensitivity of CML cells to imatinib and whether KW-2478 could overcome the imatinib resistance caused by BCR/ABL amplification. Through these experiments, we tried to discover whether KW-2478 could be a selective drug in CML therapy, especially in imatinib-resistant cells induced by BCR/ABL amplification.

## Methods

### Reagents and antibodies

KW-2478 (purity > 98%) and imatinib were purchased from Targetmol (USA). KW-2478 and imatinib were dissolved in DMSO. Anti-BCR/ABL, anti-STAT5, anti-caspase-3 and anti-PARP were all purchased from Cell Signaling Technology (USA). Bcl-XL, cleaved caspase-9, HSP90α and HSP90β were purchased from Bimake (USA). Anti-β-actin antibody was provided by Zhong Shan Jin Qiao (China). Other antibodies were provided by Huabio (Hangzhou, China).

### Cell lines and culture

The human acute myeloid cell lines THP1 and KG-1a and chronic myeloid cell lines K562, 32D, KCL-22, and SUP-B15 (Cell Bank of Shanghai Institute of Cell Biology, Chinese Academy of Sciences) were maintained in RPMI-1640 medium with 10% foetal bovine serum (Gibco, USA). All cells were maintained at 37 °C in a humidified atmosphere of 5% CO_2_. KCL-22 cells are lymphoblast-like suspension cells from chronic myeloid leukaemia patients. K562 cells are lymphoblasts isolated from the bone marrow of a chronic myeloid leukaemia patient. K562/G01, an imatinib-resistant cell line, was derived from K562 cells after months of coculture with imatinib. The 32DP-T315I cell line was derived from murine myeloid blast 32D cells stably transfected with the *BCR/AB*L fusion gene containing the T315I point mutation. SUP-B15 cells are human ALL cells containing the BCR/ABL fusion protein.

### Clinical samples

The peripheral blood of healthy individuals (2 cases) was obtained from Chongqing Medical University, China. Peripheral blood mononuclear cells were collected by mononuclear cell separation reagents (Solarbio, China). For the use of human samples, informed consent was acquired. The basic information of these two healthy individuals is shown in Additional file 1: Table S1.

### Cell viability assay

Cell proliferation was detected by CCK-8 assay. K562 cells and K562/G01 cells (5000 cells/well) were seeded in 96-well culture plates with increasing concentrations of KW-2478, followed by the addition of 10 µl of CCK-8 at selected timepoints (0–72 h). The absorbance at 450 nm was measured with a microplate reader (Eon, BioTek, USA). The drug concentration resulting in 50% inhibition of growth (IC50) was determined. The cell survival rate of each group treated with different concentrations of KW-2478 was obtained, and the IC50 was calculated with GraphPad Prism 8.0 software.

### Western blotting

Preliminary experimental results showed that KW-2478 had a stronger lethal effect on K562/G01 cells than K562 cells, so we treated K562 and K562/G01 cells with different concentrations of KW-2478 to obtain sufficient protein for western blotting. SDS-PAGE was used to separate proteins, which were subsequently transferred to PVDF membranes and blocked in 5% fat-free milk. The indicated proteins were probed at a 1:1000 dilution with matching primary antibodies, incubated with secondary antibodies and identified using ECL reagent.

### Apoptosis assay

An Annexin V-FITC Apoptosis Detection Kit (Sungene Biotech, Tianjing) was used to detect the percentage of apoptotic cells. K562 and K562/G01 cells (10^6^ cells) were treated for 48 h with KW-2478 at various doses followed by Annexin-V/PI staining. Then, cell apoptosis was detected by flow cytometry (Beckman Coulter, USA).

### Cell cycle analysis

Harvested cells were rinsed with phosphate-buffered saline (PBS), fixed in 70% ethanol, and kept for an hour at 4 °C. The cells were then stained with 500 µl of RNase/PI mixed dye solution. The cell cycle was subsequently examined by flow cytometry and analysed by CytExpert.

### Mitochondrial membrane potential assay

The mitochondrial membrane potential of K562 and K562/G01 cells was measured using the fluorescent dye JC-1. After 48 h of treatment with KW-2478, the cells were incubated with 10 µg/ml JC-1 for 25 min at 37 °C in the dark. Cells were washed with JC-1 staining buffer and detected by flow cytometry.

### Coimmunoprecipitation (Co-IP)

After 48 h of incubation with or without KW-2478 (2 µM), CML cells were collected and washed. Mouse anti-BCR/ABL antibody (Santa Cruz, USA) and rabbit anti-HSP90α antibody (Genetex, USA) were used in Co-IP assays. Protein A/G magnetic beads were purchased from MCE (USA). Pulled down proteins were detected by western blots with rabbit anti-BCR/ABL antibody (CST, USA) or rabbit anti-HSP90α antibody (Bimake, USA).

### DAPI staining assay

CML cells (10^5^ cells) were treated with or without KW-2478 for 48 h. Cells were then washed twice with ice-cold PBS, followed by fixation with 4% paraformaldehyde for 15 min. Fluorescence microscopy was used to examine the cells after they were stained with DAPI at a concentration of 5 g/ml for 15 min at 37 °C.

### Quantitative real-time PCR (qRT-PCR)

TaKaRa Bio provided the reagents and standard techniques for total RNA extraction, RNA reverse transcription into cDNA, and RT-qPCR. ACTB was used as an internal reference gene. Relative expression levels of mRNA were calculated using the 2−ΔΔCq method. The primer sequences used in this study are shown in Additional file 1: Table S2.

### Murine leukaemogenesis model

Before being injected with K562 or K562/G01 cells, 7-week-old NOD/SCID mice were given 250 cGy of X-ray radiation. Then, 5 × 10^6^ K562 or K562/G01 cells were injected into mice via the tail vein (5 mice for each group). After 7 days of inoculation, the mice successfully carrying the xenograft in the treatment groups were administered KW-2478 at a dose of 50 mg/kg/day by intraperitoneal injection, and the mice in the control groups were given the same amount of DMSO. KW-2478 was administered every other day for 3 weeks. During drug administration, the condition, weight change, and white blood cell counts of the mice were all examined weekly. The remaining surviving mice were sacrificed on Day 90 after tumour formation. The livers and spleens were weighed, and the tissues were stained with HE. The infiltration of leukaemic cells in livers, spleens or bone marrow was detected by immunofluorescence experiments. All methods in the experiment were authorized by Chongqing Medical University’s Ethics Committee and carried out in line with applicable laws and regulations.

### Statistical analysis

At least three independent experiments validated all of the findings. The results are expressed as the mean ± standard deviation. The main effect was estimated by the program GraphPad Prism 8.0 using the t test (two groups) or one-way ANOVA (more than two groups). Significance was defined with p values < 0.05 (*).

## Results

### KW-2478 is a safe HSP90α inhibitor with a BCR/ABL-specific inhibitory ability and a strong suppressive effect onimatinib-resistant CML cells

**Fig. 1:**
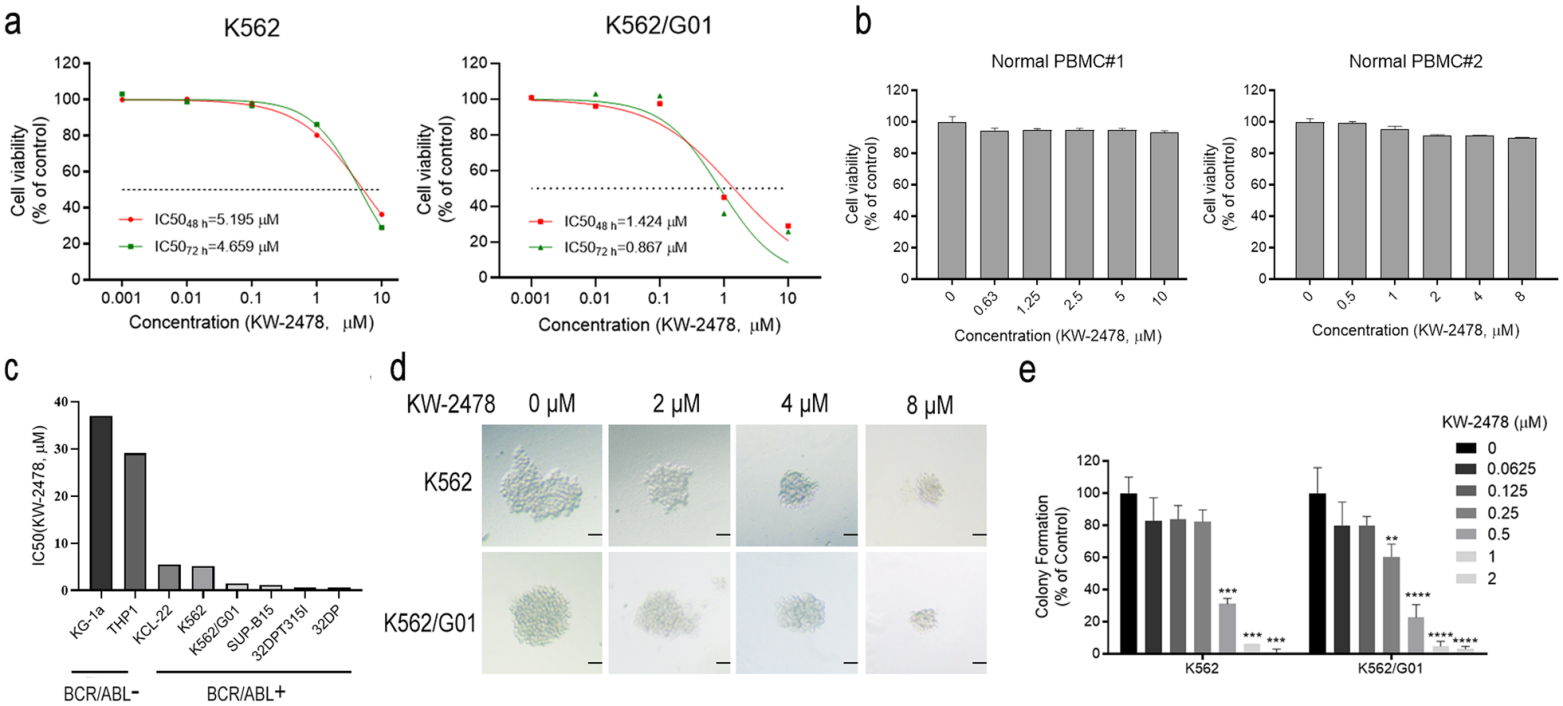
KW-2478 inhibited the growth of BCR/ABL-positive cells with no obvious effect on BCR/ABL-negative cells. **a** The effects of KW-2478 on the growth of K562 and K562/G01 cells were assessed by CCK-8 assay after incubation for 24, 48 and 72 h. **b** The survival rate of PBMCs from two healthy individuals was detected by CCK-8 assay after incubation with KW-2478 for 48 h. **c** The IC50 values of KG-1a, THP1, KCL-22, K562, K562/G01, SUP-B15, 32DP-T315I and 32DP cells were detected by CCK-8 assay after 48 h of treatment with KW-2478. **d**, **e** The proliferation of CML cells after KW-2478 treatment was detected using a colony formation assay, and the results were statistically analysed. The scale bar represents 100 μm. The values are the mean standard deviation of three independent experiments. Asterisks denote statistically significant differences compared with the control group

We detected the effect of KW-2478 on the growth of K562 and K562/G01 cells harbouring the BCR/ABL fusion protein. We discovered that KW-2478 inhibited CML cell proliferation in a dose- and time-dependent manner. The IC50 values of the KW-2478-treated K562 cells at 48 and 72 h were 5.195 µM and 4.659 µM, respectively. The IC50 values of the K562/G01 cells treated under the same conditions were 1.424 µM and 0.867 µM (Fig. 1a). We also evaluated the effect of KW-2478 on the proliferation of normal human peripheral blood mononuclear cells (PBMCs). Interestingly, KW-2478 did not have a strong inhibitory effect on the viability of normal human PBMCs, even when the concentration of KW-2478 reached 10 µM (Fig. 1b). We also calculated the IC50 values of other BCR/ABL-positive and BCR/ABL-negative cells. The IC50 values of the KG-1a, THP-1, KCL-22, K562, K562/G01, SUP-B15, 32DP and 32DP-T315I cells treated with KW-2478 for 48 h were 37.06 µM, 29.07 µM, 5.473 µM, 5.196 µM, 1.446 µM, 1.166 µM, 0.639 µM and 0.6138 µM, respectively. The results showed that the IC50 value of the BCR/ABL-negative cells (KG-1a and THP-1 cells) was much higher than that of the BCR/ABL-positive cells (KCL-22, K562, K562/G01, SUPB15, 32DP and 32DP-T315I cells). These results indicated that KW-2478 specifically inhibited BCR/ABL-positive cells (Fig. 1c). The results of the colony-forming assay showed that the number and size of colonies formed by K562 or K562/G01 cells were significantly decreased by KW-2478. We also discovered that at the same concentration of KW-2478, the inhibitory effect on colony formation in K562/G01 cells was more stronger than that in K562 cells. When K562/G01 cells were treated with 0.25 µM KW-2478, the number of colonies was significantly reduced compared with that in the control group. When the concentration of KW-2478 reached 0.5 µM, the colony number of K562/G01 cells showed a significant difference (Fig. 1d, e). Collectively, KW-2478 inhibited the growth of CML cells, and K562/G01 cells were more sensitive to KW-2478 than K562 cells. The above experiments proved that KW-2478 was a safe HSP90α inhibitor with a BCR/ABL-specific inhibitory ability and showed strong inhibition against imatinib-resistant cells.

### KW-2478 induces G2/M phase cell cycle arrest in imatinib-sensitive and imatinib-resistant CML cells

**Fig. 2:**
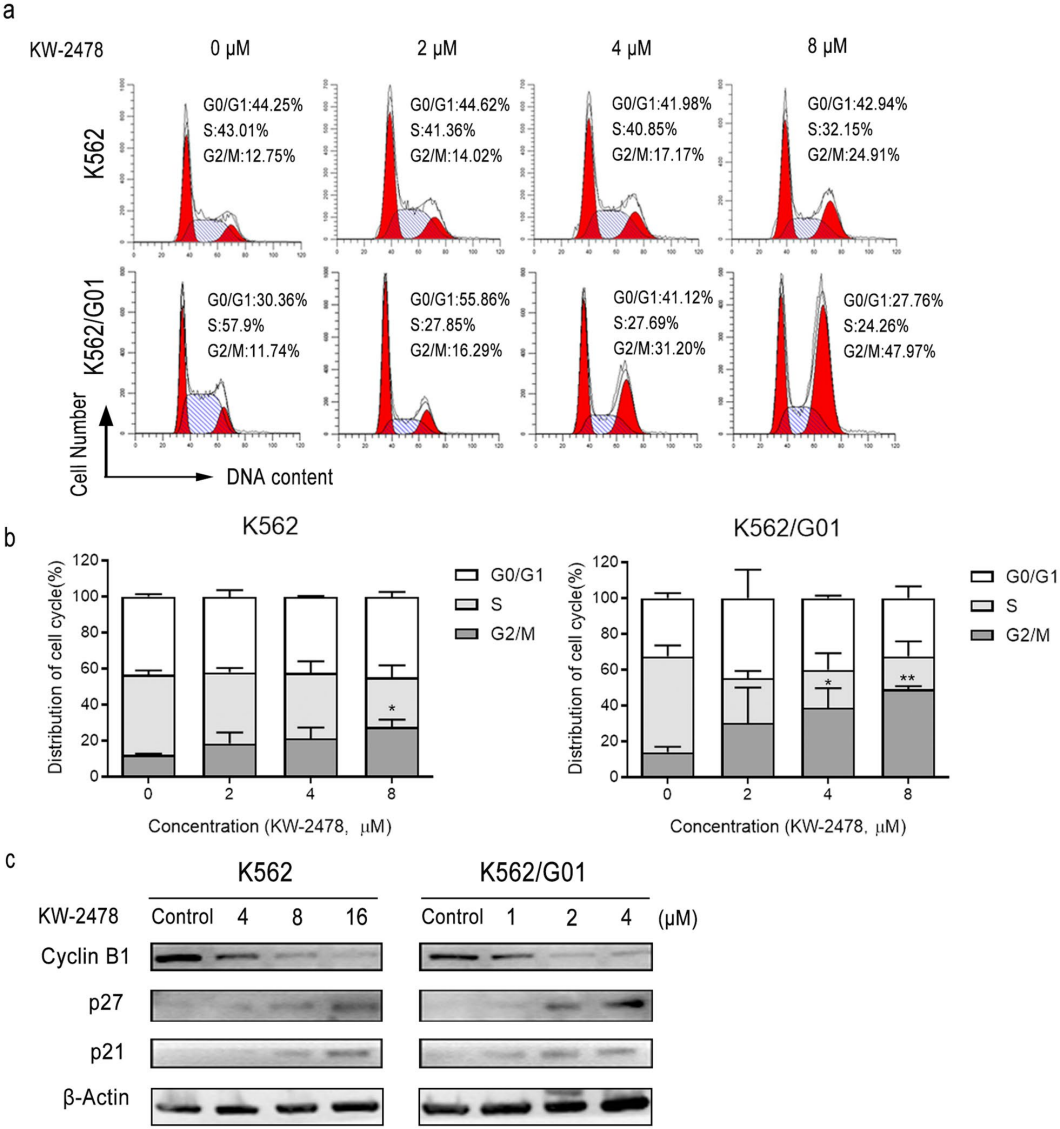
KW-2478 induced cell cycle arrest in the G2/M phase in CML cells. **a** After K562 and K562/G01 cells were treated with KW-2478 for 48 h, the cell cycle distribution was determined using PI staining and flow cytometry. **b** Following flow cytometry detection, the cell proportions in each cell cycle phase were summarized. **c** Levels of expression of cell cycle-related proteins, such as cyclin B1, p21 and p27, were quantitatively analysed by western blots. The results are expressed as the mean ± SD. *P < 0.05, **P < 0.01

The effect of KW-2478 on K562 and K562/G01 cell cycle progression was investigated by flow cytometry. The results revealed that KW-2478 induced a dose-dependent increase in the G2/M-phase population and a decrease in the S-phase population. Compared to that of the control group (12.75%), the G2/M-phase cell population of the KW-2478-treated K562 cells reached 14.02%, 17.17% or 24.91% at concentrations of 2, 4 or 8 µM, respectively. The G2/M phase arrest caused by KW-2478 was more effective in K562/G01 cells. Moreover, the K562/G01 cell population at G2/M-phase increased to 47.97% at 8 µM KW-2478 (Fig. 2a, b). To further investigate the reason for the G2/M phase arrest induced by KW-2478, we detected the expression of G2/M phase-regulating proteins by western blotting. It has been reported that activated cyclin B1/CDC2 complexes are required for progression through G2/M. Moreover, p27 and p21 can inhibit the function of the cyclin B1/CDC2 complexes and block cell cycle progression [31]. Our results showed that the expression of p21 and p27 increased, while cyclin B1 expression was reduced considerably after KW-2478 treatment for 48 h (Fig. 2c). The results in this part showed that KW-2478 induced cell cycle arrest in G2/M phase by downregulating cyclin B1 expression and upregulating p21 and p27 expression, and the G2/M phase arrest was much more significant in K562/G01 cells than in K562 cells.

### KW-2478 induces apoptosis by activating the caspase pathway in imatinib-sensitive and imatinib-resistant CML cells

**Fig. 3:**
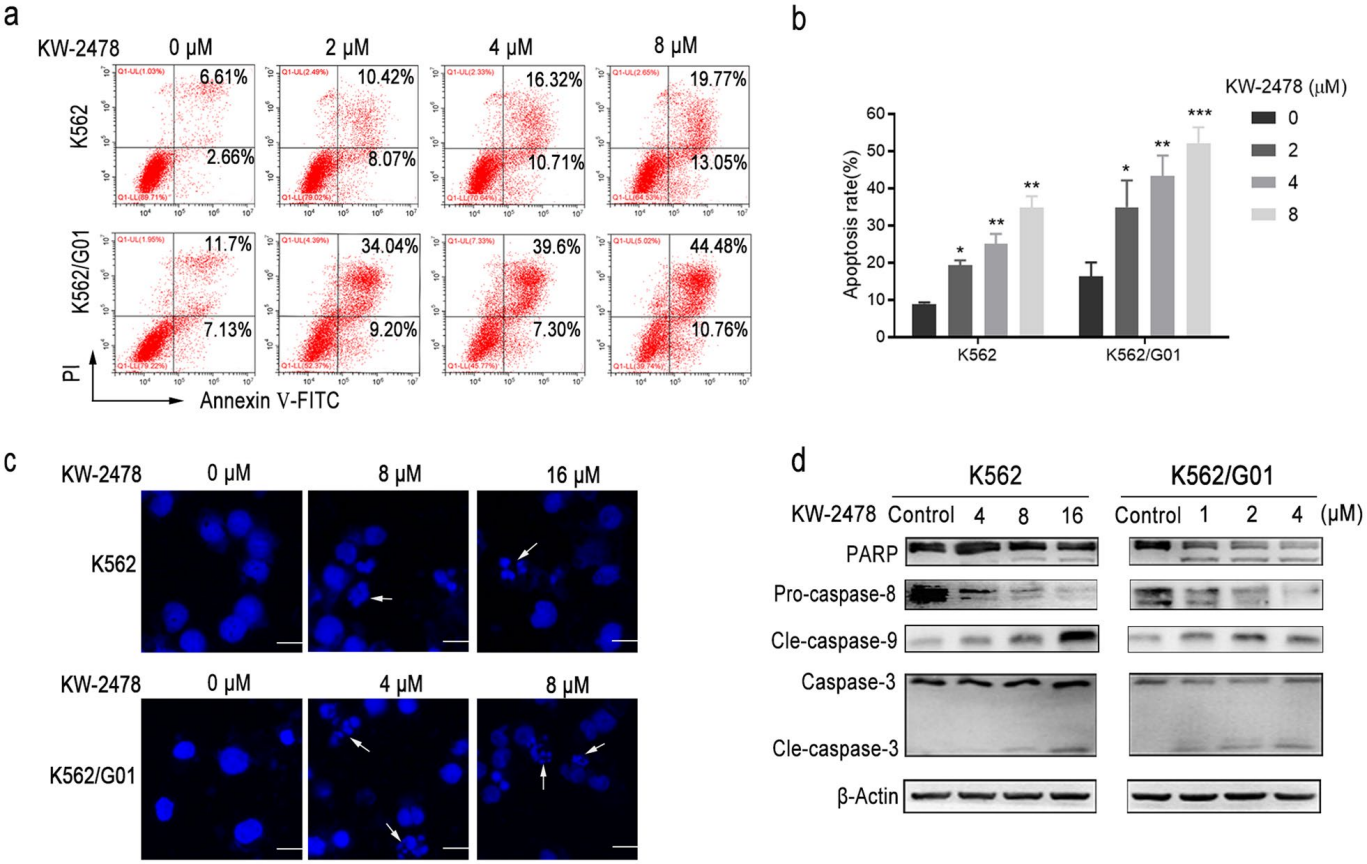
KW-2478 induced apoptosis by activating the caspase pathway in CML cells. **a** After K562 and K562/G01 cells were treated with KW-2478 for 48 h, flow cytometry was used to detect cell apoptosis after annexin-V-FITC/PI labelling. **b** The cell apoptosis rates were summarized after flow cytometry detection. **c** The morphology of apoptotic cells was examined after DAPI staining. The scale bar represents 10 μm. **d** The expression levels of cell apoptosis-related proteins, such as PARP, pro-caspase-8, cle-caspase-9 and caspase-3, were quantitatively analysed by western blots after exposure to KW-2478 for 48 h. β-Actin expression served as a loading control

Treatment with KW-2478 for 48 h resulted in an increased percentage of apoptotic cells compared to that of the control group (Fig. 3a, b). After treatment with the same dose of KW-2478, the percentage of apoptotic K562/G01 cells was substantially higher than that of K562 cells. DAPI staining was used to identify morphological alterations associated with apoptosis. CML cells were stained with DAPI dye and observed with fluorescence microscopy. In the control group, cell nuclei were round with uniform size, and chromatin was distributed homogeneously. However, chromatin condensation, marginalization and nuclear fragmentation were commonly detected in the KW-2478-treated cells (Fig. 3c). It has been reported that the activation of the caspase pathway in response to proapoptotic signals is crucial in the process of apoptosis triggered by a range of stimuli, which necessitates the activation of early caspases such caspase-8 and -9 [32]. Treatment of K562 cells with 8 µM KW-2478 and K562/G01 cells with just 1 µM KW-2478 resulted in PARP cleavage (Fig. 3d). In parallel, KW-2478 led to an increase in caspase-3 and caspase-9 cleavage in CML cells, which indicated their activation. The decrease in pro-caspase-8 levels indicated that its proapoptotic activity increased (Fig. 3d). These findings revealed that KW-2478 activated the caspase pathway and caused CML cell apoptosis, and the effect was stronger in imatinib-resistant K562/G01 cells.

### Apoptosis induced by KW-2478 results from mitochondrial dysfunction

**Fig. 4:**
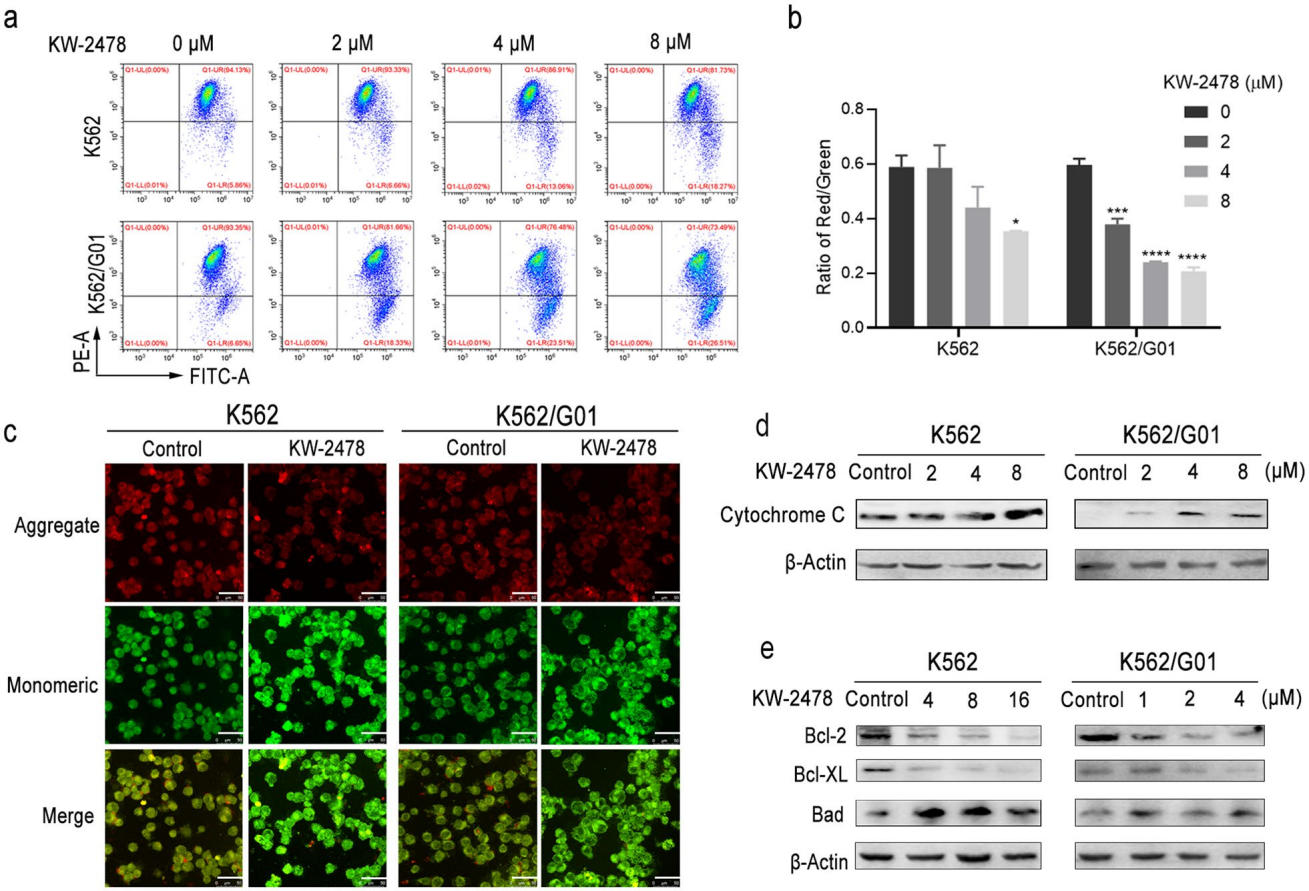
Apoptosis induced by KW-2478 is relevant to mitochondrial dysfunction. **a** The changes in mitochondrial membrane potential were detected by flow cytometry after CML cells were stained with JC-1. **b** The ratio of red fluorescence to green fluorescence was summarized after flow cytometry detection. **c** Representative images of JC-1 fluorescence imaging. The scale bar represents 50 μm. **d** Cytochrome C released from mitochondria was detected by western blot. **e** Western blotting was used to detect the expression level of the Bcl-2 protein family

Caspase activation is linked to mitochondrial malfunction and cytochrome C release into the cytoplasm. JC-1 is a fluorescent dye that characterizes the dissipation of mitochondrial membrane potential (∆Ψm) by a considerable change of red to green fluorescence [33]. KW-2478 enhanced the emission of green fluorescence in CML cells, which demonstrated the dissipation of ∆Ψm during apoptosis (Fig. 3a). The ∆Ψm of K562/G01 cells was significantly decreased after incubation with 2 µM KW-2478, whereas the ∆Ψm of K562 cells decreased significantly only when the concentration of KW-2478 reached 8 µM (Fig. 4b). Furthermore, JC-1 fluorescence imaging confirmed this finding. Compared with those of the control group, K562 and K562/G01 cells showed stronger green fluorescence and weaker red fluorescence after treatment with KW-2478, which indicated a decrease in their ∆Ψm (Fig. 4c). The effect of KW-2478 on proteins involved in mitochondrial apoptotic pathways was then investigated. We first obtained the proteins of CML cells after removal of mitochondria. The quantities of cytochrome C released into the cytoplasm from the mitochondria increased after treatment with KW-2478 (Fig. 4d). These findings indicated that KW-2478 induced apoptosis partly by disrupting the mitochondrial membrane potential. The Bcl-2 family is an important regulator in mediating the release of cytochrome C. Bad inhibits the release of cytochrome C, while Bcl-2 and Bcl-XL promote its release [34]. The protein expression of the Bcl-2 family was studied to learn more about the mechanisms of apoptosis induced by KW-2478 in CML cells. The results revealed that Bad, a proapoptotic protein, had significantly upregulated expression. The expression levels of Bcl-2 and its closest relative, Bcl-XL, were decreased in the KW-2478-treated CML cells. These effects were dose dependent, and a lower concentration of KW-2478 caused a more obvious effect in K562/G01 cells (Fig. 4e). The results showed that KW-2478 promoted the release of cytochrome C into the cytoplasm and reduced mitochondrial membrane potential by regulating Bcl-2 family proteins, thereby inducing apoptosis through the mitochondrial pathway in CML cells.

### KW-2478 inhibits the chaperone function of HSP90 and then weakens the BCR/ABL and MAPK signalling pathways

**Fig. 5:**
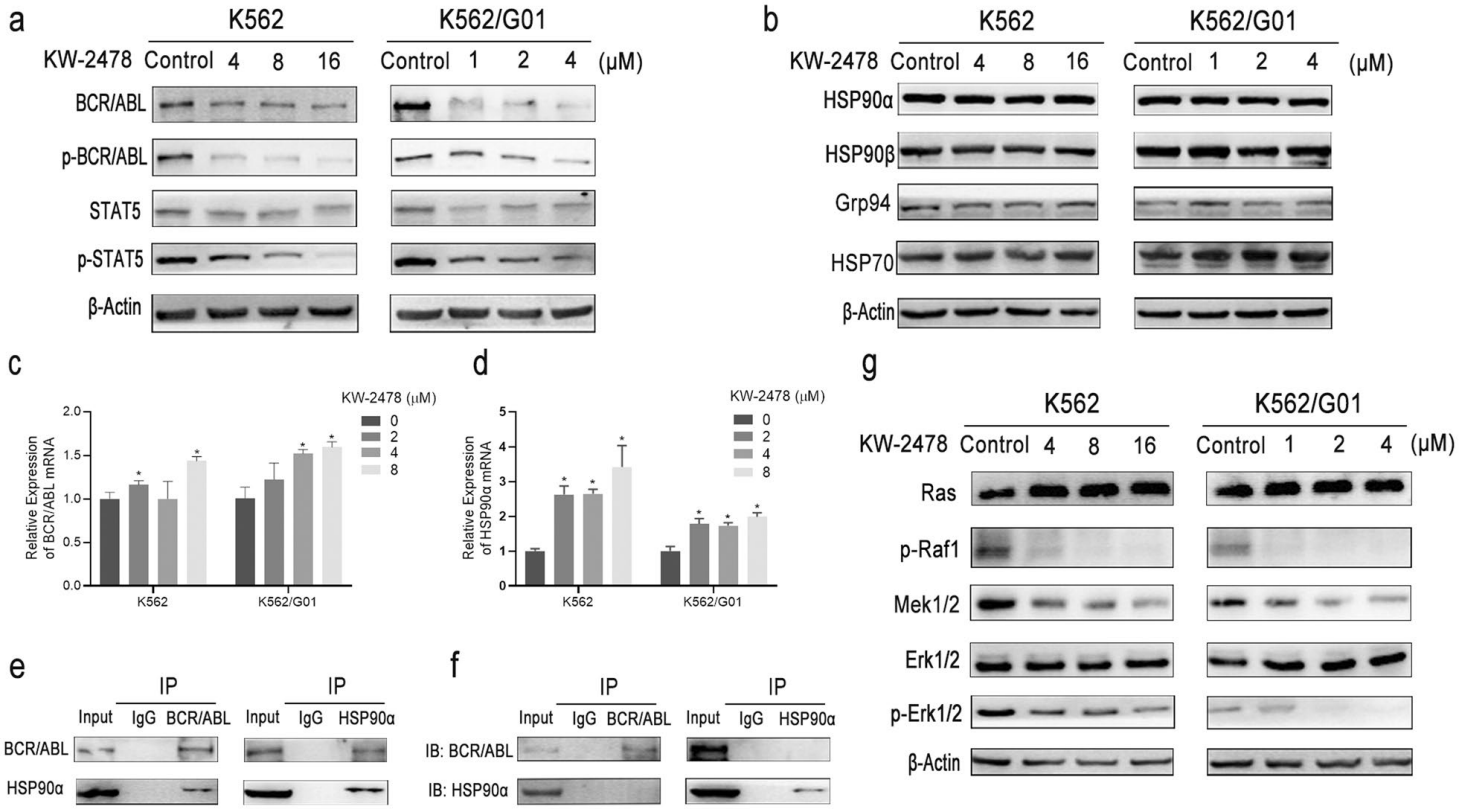
KW-2478 inhibited the chaperone function of HSP90α and then weakened the BCR/ABL and MAPK signalling pathways. **a** The expression levels of BCR/ABL, p-BCR/ABL, STAT5 and p-STAT5 in CML cells after KW-2478 treatment were detected by western blots. **b** The expression levels of HSP90α, HSP90β, Grp94 and Hsp70 in CML cells after KW-2478 treatment were quantitatively analysed by western blot. **c** The mRNA level of *BCR/ABL* was detected with qRT-PCR. β-Actin was used as an internal control. **d** The mRNA level of HSP90α was detected with qRT-PCR. **e** The interaction between HSP90α and BCR/ABL was analysed by Co-IP assays with no KW-2478 treatment. **f** The influence of KW-2478 on the interaction between HSP90α and BCR/ABL was analysed by Co-IP assays. **g** Molecules in the MAPK signalling pathway, such as Ras, phospho-Raf1, Mek1/2, phosphorylated and total Erk1/2, were analysed by western blots. The results are expressed as the mean ± SD. *p < 0.05

Our results clearly demonstrated that the levels of phospho-BCR/ABL, BCR/ABL, phospho-STAT5, and STAT5 in K562 and K562/G01 cells were diminished after KW-2478 treatment (Fig. 5a). To investigate how KW-2478 altered BCR/ABL expression, we examined the mRNA level of *BCR/ABL* in response to KW-2478. The results indicated that the mRNA level of *BCR/ABL* slightly increased, which suggested that KW-2478 affected BCR/ABL expression mainly through post-transcriptional mechanisms (Fig. 5c). The HSP90 family is an important chaperone for BCR/ABL, so we detected the effect of KW-2478 on the protein and mRNA expression of the HSP90 family. The results showed that HSP90α mRNA expression was upregulated after treatment with KW-2478, but no difference was observed in the protein levels of HSP90α, HSP90β and Grp94 (Fig. 5b, d). HSP70 is a member of the heat shock protein family and can be used as an indicator of the heat shock response (HSR) [35]. We found that HSP70 was barely affected by KW-2478, which indicated that KW-2478 did not induce a remarkable heat shock response (Fig. 5b). Next, we further investigated whether the downregulated protein expression of BCR/ABL by KW-2478 was caused by the chaperone function of HSP90α. K562/G01 cells were cultured with or without KW-2478 for 48 h, and then, the interaction between HSP90α and BCR/ABL was detected by coimmunoprecipitation. The results indicated that BCR/ABL interacted with HSP90α in the absence of KW-2478 (Fig. 5e), while the interaction between them was disturbed by KW-2478 (Fig. 5f). These experiments proved that HSP90α could bind to BCR/ABL in K562/G01 cells and that KW-2478 could obstruct their binding. Without the protection of HSP90α, BCR/ABL might degrade, which leads to the downregulation of the expression of BCR/ABL and its downstream molecules and reduced oncogenic properties of CML cells. The results of western blot experiments proved that KW-2478 did not affect the expression of Ras but significantly downregulated p-Raf1 expression. Moreover, decreased expression of Mek1/2 and p-Erk1/2 was observed after treatment with KW-2478 in CML cells (Fig. 5g). Mek1/2 and p-Erk1/2 are downstream molecules of Raf1, and all three proteins are important regulators in the MAPK pathway, which is critical for cell proliferation. These results indicated that p-Raf1 expression was directly downregulated by KW-2478 but not through Ras. Overall, KW-2478 simultaneously inhibited the BCR/ABL and MAPK signalling pathways by disturbing the chaperone function of HSP90α and then induced obvious growth suppression in CML cells.

### KW-2478 synergistically functions with imatinib to induce apoptosis and growth inhibition in imatinib-sensitive and imatinib-resistant CML cells

**Fig. 6:**
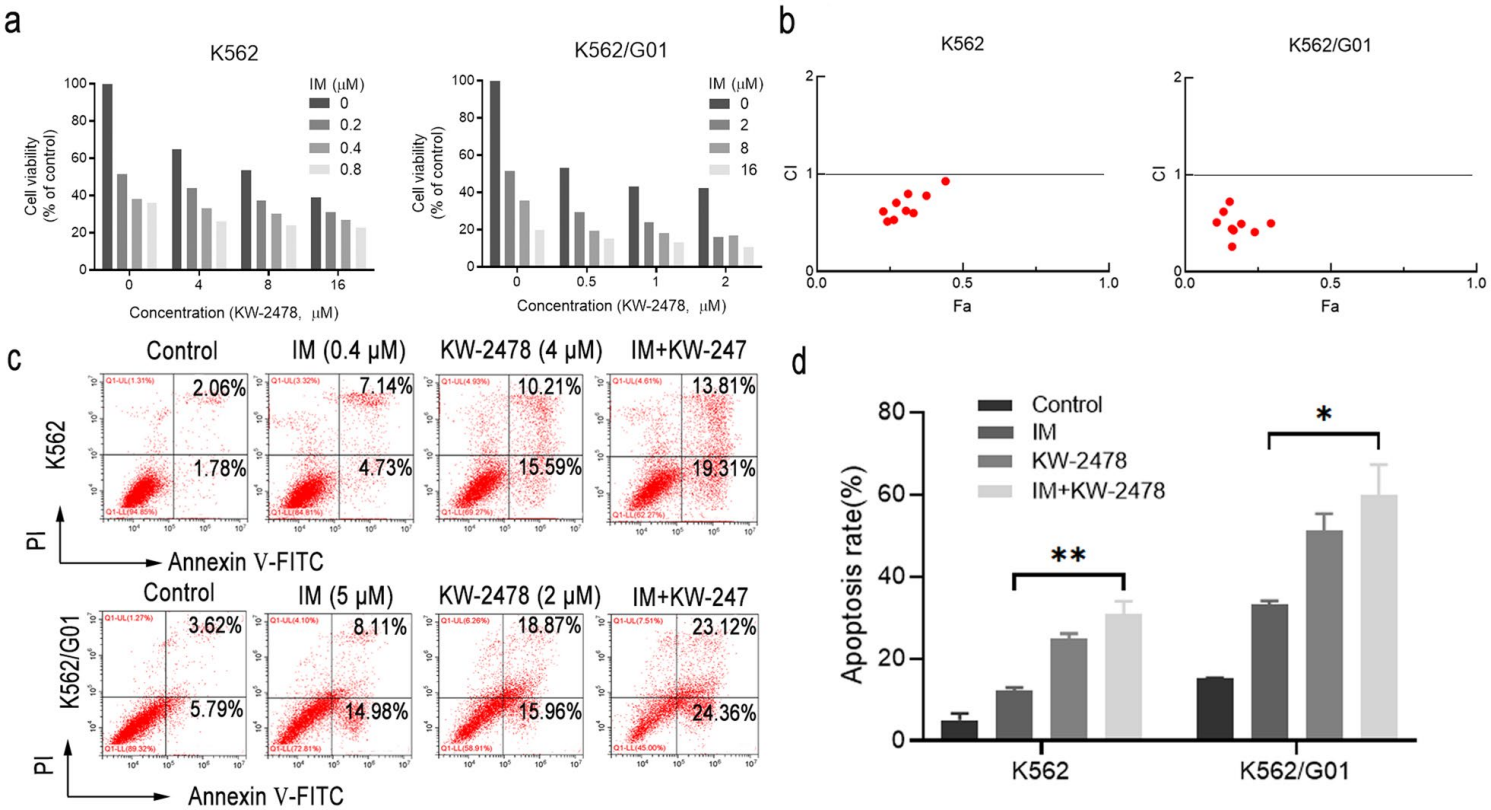
KW-2478 synergistically functioned with imatinib to induce apoptosis and growth inhibition in CML cells. **a** A CCK-8 assay was used to determine the combined effects of KW-2478 and imatinib on cell viability of K562 and K562/G01 cells. **b** The combination index of K562 or K562/G01 cells was analysed using CompuSyn software. **c** Following treatment with KW-2478, imatinib or both, cell apoptosis was detected by flow cytometry. **d** Statistical analysis of the apoptosis rates of K562 and K562/G01 cells treated with KW-2478, imatinib or both

To investigate the synergistic effect of KW-2478 with imatinib, we treated K562 cells and K562/G01 cells with KW-2478, imatinib or both for 48 h, followed by a CCK-8 assay. Considering the different sensitivities of K562 and K562/G01 cells to KW-2478 and imatinib, we used different drug combinations. The combination exhibited a higher inhibition rate on either K562 or K562/G01 cells than KW-2478 or imatinib alone (Fig. 6a). The combination index (CI) was analysed with CompuSyn software. All values of the CI were than 1 indicating that the combined growth inhibitory effect was synergistic (CI < 1, = 1, and > 1 represent synergistic, additive, and antagonistic effects, respectively) (Fig. 6b, Additional file 1: Tables S3 and S4). Moreover, the CI values of the K562/G01 groups were lower than those of the K562 groups, indicating that the synergistic effect was more effective in K562/G01 cells. Then, flow cytometry analysis was used to examine the apoptotic index values to learn more about the influence of this combination on CML cell apoptosis. As indicated in Fig. 6c, d, the combination group had a greater apoptotic rate than the KW-2478 or imatinib alone groups. These experiments showed that KW-2478 had a synergistic effect with imatinib whether in growth inhibition or induction of apoptosis. The combination of imatinib and KW-2478 is a prospective treatment to address TKI resistance and intolerance in CML treatment.

### KW-2478 has effective antitumor activity in imatinib-sensitive and imatinib-resistant CML-like mouse models

**Fig. 7:**
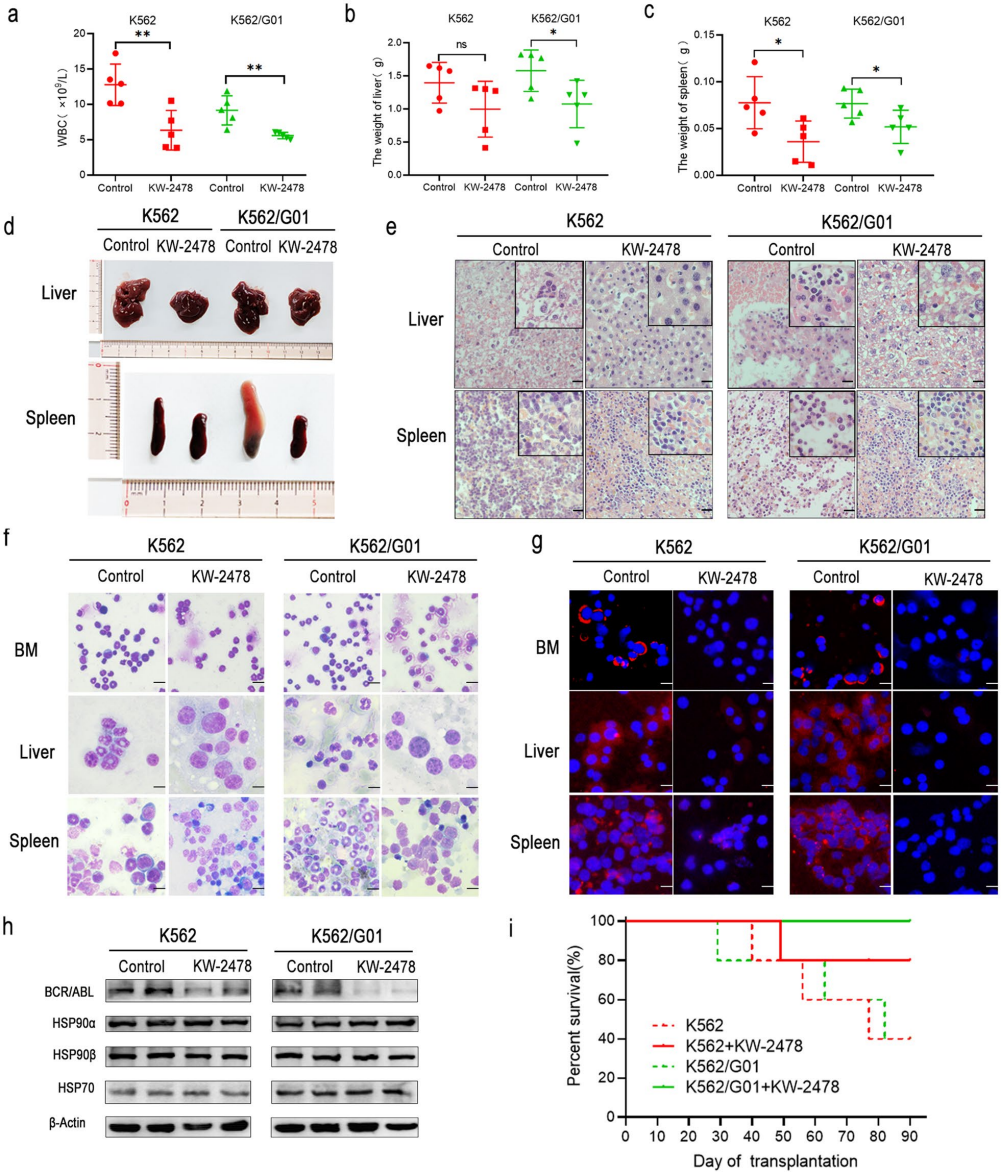
KW-2478 had effective antitumor activity in imatinib-sensitive and imatinib-resistant CML-like mouse models. **a** The maximum WBC counts of mice were recorded. **b**, **c** The liver and spleen weights of mice were measured. The results are expressed as the mean ± SD. *p < 0.05, **p < 0.01. **d** Images of the liver and spleen are displayed. **e** Murine liver and spleen infiltration was analysed by HE staining. The scale bar represents 50 μm. **f** Cells from mouse bone marrow, liver, and spleen were stained by Wright’s stain and then photographed under a microscope. The scale bar represents 20 μm. **g** The expression of BCR/ABL in each tissue was detected by immunofluorescence assays. The scale bar represents 10 μm. **h** The expression levels of BCR/ABL and HSP90 family proteins from mouse bone marrow cells were detected by western blots. **i** The survival curves of mice were analysed by the Kaplan–Meier method

We confirmed that KW-2478 had an excellent antileukaemia effects in vitro, so we further investigated its antileukaemia effect in vivo. The in vivo antitumor activity of KW-2478 was examined in a NOD-SCID mouse model bearing human CML xenografts. We generated two CML mouse models. One was injected with K562 cells to mimic ordinary CML disease, and the other was injected with K562/G01 cells to mimic imatinib-resistant CML disease. The results showed that the mice that received KW-2478 treatment displayed lower white blood cell counts than the controls in both models (Fig. 7a). When the mice showed obvious symptoms, such as weight loss, listlessness, vertical hair and arched back or unstable gait, we sacrificed the mice. The spleen and liver were removed and weighed. As shown in Fig. 7b–d, the mice in the control groups had more severe hepatosplenomegaly than the mice in the KW-2478-treated groups. The infiltration of leukaemic cells in the liver and spleen was examined by HE staining and Wright’s staining. The results of HE staining revealed that the number of rod-shaped leukocytes and lobulated leukocytes that infiltrated the liver and spleen significantly decreased in the KW-2478 treatment groups compared to the control groups (Fig. 7e). The results of Wright’s staining showed less active proliferation in the bone marrow and less granulocytes infiltrated in the liver and spleen in the KW-2478-treated groups than in the control groups (Fig. 7f). We also detected the expression of BCR/ABL in cells from mouse livers, spleens and bone marrow to further confirm the infiltration of CML cells by immunofluorescence assays. We found that a high level of BCR/ABL expression was detected in these tissues from control group mice, while we barely detected the expression of BCR/ABL in the KW-2478-treated groups (Fig. 7g). We extracted bone marrow proteins from these mice and performed western blotting to detect the expression of BCR/ABL and HSP90 family proteins. The BCR/ABL protein level in the KW-2478-treated mice was lower than that in the control mice. There was no obvious difference in the protein levels of the HSP90 family and HSP70 between the KW-2478-treated groups and the control groups (Fig. 7h). As shown in Kaplan–Meier survival curves, the mouse survival time in the KW-2478 groups was prolonged. In the observation period of 90 days, 40% of the mice survived in the K562 control group, while 80% of mice survived in the KW-2478 treatment group. In addition, 40% of the mice survived in the K562/G01 control group, while 100% of the mice survived in the KW-2478 treatment group (Fig. 7i). Moreover, the remaining mice in the two control groups still showed CML, while the mice in the two KW-2478 treatment groups showed no signs of disease. We predicted that the control mice would die before the treatment mice if the observation period was extended.

In general, these results proved that KW-2478 prolonged the mouse lifespan and alleviated disease symptoms and tumour cell infiltration in both the K562 and K562/G01 mouse models.

## Discussion

CML is a myeloproliferative tumour defined by a reciprocal chromosomal translocation t(9;22)(q34;q11) [11]. However, despite significant advances in TKI treatment, 30–50% of patients experience failure of frontline imatinib therapy after 5 years [36]. Small molecule inhibitors targeting cellular oncoproteins are approved for CML malignancies [37]. HSP90 acts on a broad repertoire of client proteins that play a vital role during tumorigenesis [38]. BCR/ABL is one of the crucial client proteins of HSP90. Many oncoproteins expressed in mutant forms, such as BCR/ABL, rely on HSP90 for stability and activity far more than their normal counterparts. BCR/ABL amplification has been reported to be an important mechanism of TKI resistance [39]. Therefore, targeting HSP90 with its inhibitors would be a prospective therapy for CML treatment and TKI-resistant patients.

**Fig. 8:**
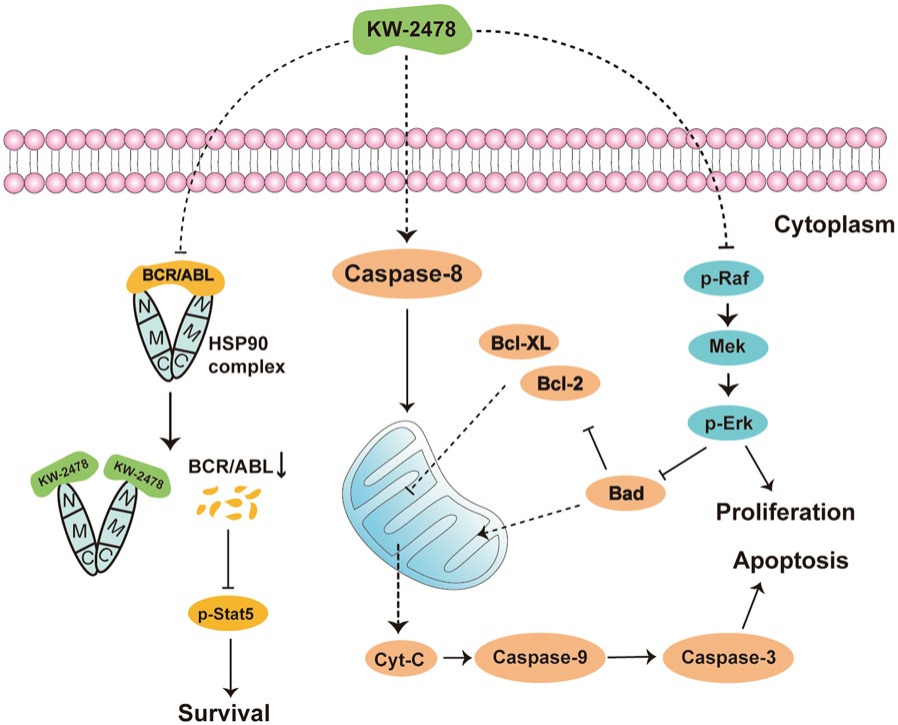
Schematic diagram of the function and mechanisms of KW-2478 in CML cells. KW-2478 degrades BCR/ABL and downstream signalling proteins by inhibiting the binding of HSP90α and BCR/ABL. This molecule inhibits the growth of CML cells by inhibiting the MAPK signalling pathway. KW-2478 causes cytochrome C release and mitochondrial dysfunction to activate the caspase pathway and then induces apoptosis in CML cells

In this study, we selected KW-2478 as a potential means to resolve the problem of CML treatment. KW-2478 is a novel HSP90 inhibitor that has good water solubility, safety and antitumor effects in other malignant diseases [40]. We demonstrated that KW-2478 is a safe HSP90α inhibitor with BCR/ABL-specific inhibitory ability. This molecule inhibits the chaperone function of HSP90α and then weakens the BCR/ABL and MAPK signalling pathways to inhibit CML cell growth. KW-2478 regulates some cell cycle-related proteins and then induces G2/M phase arrest. It causes mitochondrial dysfunction to activate the caspase pathway and then induces apoptosis in CML cells (Fig. 8).

Our results showed that KW-2478 upregulated the mRNA level of BCR/ABL but downregulated its protein level. We confirmed that KW-2478 directly downregulated BCR/ABL protein expression by inhibiting HSP90α chaperone function. Therefore, we hypothesized that the upregulation of BCR/ABL mRNA expression might be due to negative feedback after the decrease in BCR/ABL protein. We also found that HSP90α mRNA expression was upregulated after treatment with KW-2478, but no difference was observed in the protein levels of HSP90α, HSP90β, Grp94 and HSP70. The Co-IP results demonstrated that KW-2478 exerted its antitumor effect by affecting the binding of HSP90α to BCR/ABL. Hence, we speculated that KW-2478 affected only the function of HSP90α but not its protein level. The upregulation of HSP90α mRNA expression may be caused by the negative feedback of the inhibition of HSP90α function to adapt to the cell requirement for HSP90α. KW-2478 does not downregulate Ras protein expression but can significantly downregulate p-Raf1 expression and its downstream MAPK signalling pathway. Raf-1 is also one of the important client proteins of HSP90. Therefore, it is possible that KW-2478 inhibits the function of HSP90α and thus inhibits p-Raf1 expression. This hypothesis needs to be further verified by subsequent experiments.

Some studies reported that Hsp90 inhibition in combination with ibrutinib may be an option for initial treatment in CLL to prevent the outgrowth of a resistant clone in patients [41]. Therefore, we hypothesized that inhibition of HSP90 combined with IM therapy could further inhibit CML disease progression. We proved that KW-2478 has a synergistic effect with imatinib in terms of both growth inhibition and induction of apoptosis. KW-2478 has effective antitumor activity to prolong mouse lifespan and alleviate disease symptoms and tumour cell infiltration in CML-like mouse models.

Notably, KW-2478 had better antileukaemia effects in K562/G01 cells than in K562 cells. We demonstrated that KW-2478 had a stronger inhibitory ability against K562/G01 cells. The G2/M phase arrest was much more significant, and the apoptotic induction effect was stronger in K562/G01 cells. Moreover, the synergistic effect of KW-2478 with imatinib was more effective in the K562/G01 cell line. Hence, the combination of KW-2478 and imatinib would be a possible strategy to overcome the challenge of TKI resistance. However, the exact mechanism by which KW-2478 functions more effectively in K562/G01 cells remains to be further illustrated. BCR/ABL amplification is the reason for imatinib resistance in K562/G01 cells. Therefore, we speculated that K562/G01 cells were more dependent on the chaperone function of HSP90 than K562 cells. Therefore, K562/G01 cells were more significantly affected when KW-2478 inhibited the chaperone function of HSP90α in these two cell lines. However, more direct evidence is needed to verify our speculation.

In general, with the clinical application of TKIs, major progress has been made in CML therapy. Overall survival and disease-free survival have been significantly prolonged. The main challenge is to resolve TKI resistance, intolerance and lifetime dependence. The experiments presented in this study have proven that KW-2478 can efficiently depress the malignancy of imatinib-sensitive and imatinib-resistant CML cells in vitro and in vivo and effectively enhance the sensitivity of these cells to imatinib. These results address the TKI resistance caused by BCR/ABL amplification and TKI intolerance. The discovery of imatinib is a milestone for cancer therapy with targeted small-molecule inhibitors. Our study confirms that targeting the accessory protein that is vital for the function of oncoproteins is a complementary therapy with good prospects.

## Conclusions

Our study proved the anticancer effect of KW-2478 on imatinib-sensitive and imatinib-resistant CML cells by inhibiting the chaperone function of HSP90α, weakening the BCR/ABL and MAPK signalling pathways and causing mitochondrial dysfunction to activate the caspase pathway. We also demonstrated that KW-2478 had the potential to improve CML cell susceptibility to imatinib. These experiments provide a research basis for KW-2478 as a solution for TKI-resistant patients with BCR/ABL amplification and TKI-intolerant patients.

## Supplementary Information

**Additional file 1: Table S1.** Personal information about the normal human blood samples used in this study. **Table S2.** Sequences of primers used in the study. **Tables S3.** Synergy index of different combinations of KW-2478 and IM on K562 cells. **Table S4.** Synergy index of different combinations of KW-2478 and IM concentrations on K562/G01 cells.

## Data Availability

The datasets used and/or analysed during the current study are available from the corresponding author on reasonable request.

## Abbreviations

CML: Chronic myeloid leukaemia
HSP90: Heat shock protein 90
TKIs: Tyrosine kinase inhibitors
HSP70: Heat shock protein 70
17-AAG: 17-Allamin-17-demethoxygoldamycin
SD: Standard deviation
CI: Combination index
PBMCs: Peripheral blood mononuclear cells
WBC: White blood cell
