# Supplementary material for: The HSP90 inhibitor KW-2478 depletes the malignancy of BCR/ABL and overcomes the imatinib-resistance caused by BCR/ABL amplification
Source: Exp Hematol Oncol. 2022 May 27;11:33. doi: 10.1186/s40164-022-00287-w (PMC9137153; doi:10.1186/s40164-022-00287-w)
Supplement: Supplementary file 1 — Additional file 1: Table S1. Personal information about the normal human blood samples used in this study. Table S2. Sequences of primers used in the study. Tables S3. Synergy index of different combinations of KW-2478 and IM on K562 cells. Table S4. Synergy index of different combinations of KW-2478 and IM concentrations on K562/G01 cells. [file 40164_2022_287_MOESM1_ESM.docx]

**Table S1. Normal individuals information.**

| Samples | Diagnosis | Gender | Age |
| --- | --- | --- | --- |
| Nomal1 | healthy | Female | 26 |
| Nomal2 | healthy | Male | 24 |

## Table S2. Sequences of primers used in the study

| Gene | Forward/reverse | Primer Sequence(5′–3′) |
| --- | --- | --- |
| BCR/ABL | F | GCTCTATGGGTTTCTGAATGTC |
|  | R | TGGCGTGATGTAGTTGCTTG |
| HSP90α | F | CCAGTTCGGTGTTGGTTTTTAT |
|  | R | TCCTTTATTCTTCGTTCCTCCA |
| ACTB | F | ACTTAGTTGCGTTACACCCT |
|  | R | TGTCACCTTCACCGTTCC |

**Table S3. Synergy index of different combinations of KW-2478 and IM concentrations on K562 cells**

| Dose IM (μM) | Dose KW-2478 (μM) | Effect | CI |
| --- | --- | --- | --- |
| 0.2 | 4 | 0.44 | 0.92567 |
| 0.2 | 8 | 0.3748 | 0.77702 |
| 0.2 | 16 | 0.3122 | 0.79616 |
| 0.4 | 4 | 0.3301 | 0.59995 |
| 0.4 | 8 | 0.3045 | 0.62309 |
| 0.4 | 16 | 0.2712 | 0.70448 |
| 0.8 | 4 | 0.2632 | 0.52916 |
| 0.8 | 8 | 0.241 | 0.51318 |
| 0.8 | 16 | 0.2268 | 0.61643 |

**Table S4. Synergy index of different combinations of KW-2478 and IM concentrations on K562/G01 cells**

| Dose IM (μM) | Dose KW-2478 (μM) | Effect | CI |
| --- | --- | --- | --- |
| 4 | 0.5 | 0.294 | 0.49972 |
| 4 | 1 | 0.2384 | 0.40893 |
| 4 | 2 | 0.161 | 0.25892 |
| 8 | 0.5 | 0.1927 | 0.49357 |
| 8 | 1 | 0.166 | 0.42832 |
| 8 | 2 | 0.16 | 0.44325 |
| 16 | 0.5 | 0.1523 | 0.72167 |
| 16 | 1 | 0.1315 | 0.61965 |
| 16 | 2 | 0.1083 | 0.50933 |
